# Coughing correlates: insights into an innovative study using cough peak expiratory flow to predict extubation failure

**DOI:** 10.1186/s13054-016-1574-0

**Published:** 2016-12-06

**Authors:** Chuan Jiang, Antonio M. Esquinas, Bushra Mina

**Affiliations:** 1 Department of Medicine, Division of Nephrology, Northwell Health, Lenox Hill Hospital, Nephrology Division, 100 East 77th Street 3rd Floor, New York, NY 11075-1850 USA; 2 Intensive Care and Non-invasive Ventilatory Unit, Hospital Morales Meseguer, Murcia, Spain; 3 Department of Medicine, Division of Pulmonary and Critical Care Medicine, Northwell Health, Lenox Hill Hospital, New York, NY 10065 USA

**Keywords:** Weaning from mechanical ventilation, Respiratory monitoring, Respiratory failure, Chronic obstructive pulmonary disease (COPD)

## Main text

We read the innovative study by Duan et al. [1] with great interest. However, key results need to be interpreted carefully to reach the proper conclusions. First, their primary finding that patients with low cough peak expiratory flow (CPEF) have significant benefit from non-invasive positive pressure ventilation (NIPPV) in the prevention of re-intubation and 90-day mortality is not unsurprising given how CPEF represents the severity of underlying respiratory pathology. In addition, the strength of their study lay in the methodology. Each precise detail regarding the protocol of weaning and re-intubation mirrors that of previous landmark studies [2, 3]. These careful design choices help to bridge the methodological differences and heterogeneity among preceding studies.

However, their non-standardized use of CPEF cutoffs makes external validity difficult to achieve. Previous studies have studied extubation failure at various CPEF cutoffs (e.g., ≤35 L/min in Beuret et al. [4] and ≤60 L/min in Salam et al. [5] and ≤70 L/min in Duan et al. [1]). Consequently, it is not possible to determine if a subgroup of patients within the weak cough group may have derived more benefit from NIPPV. Conversely, this arbitrary cutoff may have obscured a beneficial effect of NIPPV among patients with strong coughs. This design choice segregates the two arms asymmetrically in that the baseline demographics of patients above the CPEF cutoff appear to be younger, have a higher Glascow Goma Scale (GCS) score, and lower Acute Physiology and Chronic Health Evaluation II (APACHE II) scores. Attributing their strong results to cough strength becomes a difficult proposition in light of these potential confounders.

In selective cases, we have utilized non-invasive measurements, such as the occlusion pressure at 100 ms (P0.1) and maximum inspiratory pressure (MIP), to assess respiratory drive and neuromuscular strength, respectively, in the evaluation of weaning. We have used these parameters for patients with more severe obstructive disease (i.e., lower FEV1 at baseline). Future studies in the field of CPEF utilization may opt to integrate these parameters.

Nevertheless, this positive study by Duan et al. is a promising step in establishing the role of CPEF in the algorithm for post-extubation care. The appeal of CPEF is readily apparent to the intensive care community. Its ease, ubiquity, portability, and reproducibility make it an ideal adjunctive tool in the management of post-extubation patients. We eagerly await the subsequent follow up studies from Duan and colleagues.

## Abbreviations

CPEF: Cough peak expiratory flow
NIPPV: Non-invasive positive pressure ventilation.
